# Robotic-Assisted Tubal Reanastomosis After Sterilization in the IVF Era: A Narrative Review

**DOI:** 10.3390/medicina62061054

**Published:** 2026-05-29

**Authors:** Dimitrios Papageorgiou, Vasilios Pergialiotis, Ioannis K. Papapanagiotou, Eleftherios Zachariou, Nikolaos Plevris, Savvas Petrogiannis, Nikolaos Salakos, Stylianos Kykalos, Konstantinos Kontzoglou

**Affiliations:** 1 Department of Gynecology, Athens Naval and Veterans Hospital, 11521 Athens, Greece; 2 Laboratory of Experimental Surgery and Surgical Research “N.S. Christeas”, School of Medicine, National and Kapodistrian University of Athens, 11527 Athens, Greece; 3 1st Gynaecology Department, Division of Robotic and Laparoscopic Surgery, Metropolitan General Hospital, 14232 Athens, Greece; 4 1st Department of Obstetrics and Gynecology, National and Kapodistrian University of Athens, 10679 Athens, Greece; 5 Laboratory of Anatomy, Faculty of Nursing, National and Kapodistrian University of Athens, 11527 Athens, Greece; 6 School of Medicine, National and Kapodistrian University of Athens, 11527 Athens, Greece; 7 Second Department of Propaedeutic Surgery, “Laikon” General Hospital, School of Medicine, National and Kapodistrian University of Athens, 11527 Athens, Greece

**Keywords:** robotic-assisted reanastomosis, tubal sterilization reversal, tubal anastomosis, in vitro fertilization (IVF), fertility restoration, reproductive surgery, fallopian tubes, da Vinci Surgical System, microsurgery

## Abstract

*Background and Objectives*: Robotic-assisted tubal reanastomosis (RATR) remains a clinically relevant option for selected women seeking fertility after sterilization. In the era of IVF, surgical reversal continues to be discussed because it may restore the possibility of spontaneous conception rather than offering only cycle-dependent treatment. However, the available evidence on RATR is heterogeneous and derives predominantly from observational studies. The aim of this narrative review with a structured literature search was to synthesize the published evidence on the operative, reproductive, and economic outcomes of RATR and to contextualize its role in contemporary fertility counseling. *Materials and Methods*: A structured literature search of PubMed/MEDLINE, Scopus, and Google Scholar was performed from database inception to 20 December 2025. Data were synthesized descriptively without meta-analysis. Primary robotic clinical studies were interpreted separately from secondary and contextual publications. *Results*: In total, 16 studies were included in our study. The evidence base comprised predominantly retrospective cohorts and case series; no randomized controlled trials were identified. Reported tubal patency endpoints in robotic cohorts ranged from 81.0% to 94.1%, although denominators differed across studies and were reported either per patient or per tube. Reported pregnancy outcomes ranged from 25% to 80%, reflecting substantial heterogeneity in patient selection, follow-up duration, and outcome definitions. When woman-level delivery or live-birth outcomes were explicitly reported, they were generally encouraging in selected series, although not uniformly defined. Estimated blood loss was usually minimal when reported, and conversion to laparotomy was uncommon. Any comparison with IVF was indirect because no contemporary head-to-head comparative studies were identified. Economic data were sparse, institution-specific, and methodologically heterogeneous. *Conclusions*: Available observational evidence suggests that RATR is a feasible minimally invasive option for fertility restoration in carefully selected women after sterilization. However, the evidence base remains limited by retrospective design, small cohorts, heterogeneous outcome reporting, variable follow-up, and indirect comparison with IVF. RATR should be considered within individualized fertility counseling rather than as a universal alternative to IVF. Prospective comparative studies with standardized outcome definitions, transparent reporting of prognostic factors, and robust economic evaluation are needed.

## 1. Introduction

### 1.1. Background

Female sterilization remains widely used worldwide and is estimated to meet the contraceptive needs of approximately 220 million women of reproductive age [1]. In the United States, 10.2% of women who undergo sterilization (range: 6.7–12.6%, depending on age at sterilization) later report regret and seek fertility restoration [2]. Regret is most commonly associated with changes in marital status, reproductive intentions, or the loss of a child [2]. Fertility restoration after sterilization relies on tubal recanalization using advanced microsurgical techniques to re-establish tubal continuity and patency [3]. Conventional microsurgical reanastomosis via minilaparotomy or laparotomy can achieve high pregnancy and live-birth rates, but it requires specialized expertise and operative magnification and is associated with longer recovery [4].

The development of assisted reproductive technologies (ART), and particularly in vitro fertilization (IVF), has transformed infertility care by enabling conception without the functional presence of fallopian tubes. Despite widespread adoption, IVF is not universally applicable. Treatment typically entails substantial financial costs, repeated hormonal stimulation, and procedural burdens, while live-birth rates per cycle are strongly age-dependent and commonly reported in the range of approximately 25–45% in relevant populations [5,6]. For many women—particularly younger patients with adequate tubal remnants and no additional infertility factors—restoration of natural fertility through surgical reanastomosis remains an appealing option [7].

Robotic-assisted laparoscopic microsurgery has expanded the technical capabilities of minimally invasive tubal reconstruction. Computer-aided platforms (e.g., the da Vinci Surgical System; Intuitive Surgical, Sunnyvale, CA, USA) provide high-definition 3D visualization, motion scaling, and tremor filtration, facilitating fine dissection and microsuturing within confined spaces [8,9]. Early human experience with robotic-assisted tubal recanalization was reported by Falcone et al. in 2000 using the ZEUS Surgical System (Computer Motion, Goleta, CA, USA) [10], followed by additional early feasibility reports supporting the application of robotics to reproductive microsurgery [9]. These developments enabled the evolution of robotic-assisted tubal reanastomosis (RATR) as a minimally invasive adaptation of microsurgical fertility restoration. More recently, efforts have focused on procedural standardization, including a ten-step protocol proposed for RATR [11].

### 1.2. Why Revisit Tubal Recanalization in the Era of IVF?

The global adoption of IVF has not eliminated the role of surgical fertility restoration; rather, it has reframed its indications. IVF can shorten time to pregnancy and is independent of tubal anatomy, making it the preferred option when tubes are absent, in advanced maternal age, or when male-factor infertility is present [5,6]. By contrast, tubal reanastomosis can offer competitive live-birth outcomes in younger women with favorable tubal anatomy and may reduce the cumulative financial and psychological burden associated with repeated ART cycles [12]. Cost-effectiveness and cost–benefit analyses generally suggest that surgical reversal—performed via minimally invasive or robotic approaches—tends to be more cost-effective than IVF in women below approximately 38–40 years, partly because a single successful reversal can allow multiple spontaneous pregnancies over time [13,14].

Robotic systems may enhance the feasibility of minimally invasive tubal reanastomosis by addressing technical limitations of conventional laparoscopy. Stable magnified visualization and tremor filtration can facilitate delicate mucosa-to-mucosa approximation and potentially improve reproducibility of microsuturing and patency restoration [7,10,15]. Improved ergonomics may also reduce surgeon fatigue and may facilitate dissemination of reconstructive microsurgical techniques within robotic programs, although training requirements remain substantial [11].

Importantly, RATR and IVF should be conceptualized as complementary components of post-sterilization fertility care rather than mutually exclusive alternatives. RATR aims to restore the possibility of natural conception, which may be particularly relevant for patients who desire reproductive autonomy and potentially multiple future pregnancies. IVF provides a standardized pathway that can bypass tubal factors and address additional infertility indications when present [5,6,12].

Within this framework, counseling should be patient-centered and evidence-based, explicitly incorporating age, tubal anatomy and expected remnant length, additional infertility factors, local expertise and access, and patient preferences regarding treatment burden and time to pregnancy [7,16].

### 1.3. Aims of the Review

Despite growing interest in RATR, the evidence base remains limited. Most published studies are retrospective or small prospective observational series, outcome definitions are not standardized, and comparisons with IVF are largely indirect. The aim of this narrative review with a structured literature search was to synthesize the available evidence on robotic-assisted tubal reanastomosis after sterilization, with emphasis on operative outcomes, reproductive outcomes, patient-selection considerations and economic data. A further objective was to clarify the current role of RATR in the IVF era while explicitly acknowledging the limitations imposed by heterogeneous study designs, indirect comparisons and the predominance of observational evidence.

## 2. Materials and Methods

### 2.1. Study Design

This study was designed as a narrative review with a structured literature search. A narrative synthesis was selected because the available literature on robotic-assisted tubal reanastomosis is methodologically heterogeneous and includes feasibility studies, case series, retrospective and prospective non-randomized cohorts, comparative observational studies, systematic reviews, narrative reviews, and technical reports. The narrative approach allowed inclusion of heterogeneous study designs to provide historical continuity and interpretive depth. To improve transparency and reproducibility, prespecified eligibility criteria, independent screening, and a PRISMA flow diagram were incorporated. However, the review was not designed as a formal systematic review or meta-analysis.

### 2.2. Search Strategy

A structured literature search of the PubMed/MEDLINE, Scopus and Google Scholar databases was performed. The last electronic search was conducted on 20 December 2025, using combinations of the following MeSH and free-text terms: “robotic-assisted tubal reanastomosis”, “robotic tubal anastomosis”, “robot-assisted tubal reversal”, “robotic microsurgery”, “fertility restoration,” “tubal reconstruction,” and “tubal sterilization reversal”. The search included all relevant variants through the application of Boolean operators (“AND” and “OR”). The search filters restricted results to human studies conducted in English language peer-reviewed journals. In addition, the reference lists of all eligible publications were hand-searched independently by three reviewers (E.Z., N.P. and I.K.P.). The full database-specific search strategies are presented in Supplementary File S1.

### 2.3. Study Selection and Eligibility Criteria

Titles and abstracts were screened independently by three reviewers (E.Z., N.P. and I.K.P.), followed by full-text assessment of potentially eligible publications. Disagreements were resolved by discussion and consensus.

Primary clinical studies were eligible if they included adult women undergoing robotic-assisted tubal reanastomosis after sterilization and reported at least one clinically relevant outcome, including operative time, blood loss, complications, tubal patency, pregnancy, delivery/live birth, ectopic pregnancy, or cost-related outcomes.

Because the aim of the review was not limited to direct clinical outcomes but also included historical development, technical evolution, and clinical positioning of RATR in the IVF era, selected secondary publications were also retained for contextual interpretation. These included systematic reviews, meta-analyses, narrative reviews, and technical standardization reports. These publications were analyzed separately from the primary robotic cohorts and were not treated as independent primary evidence of effectiveness.

Experimental animal research studies and conference abstracts without peer-reviewed full texts were excluded. Non-English publications and reports addressing robotic adnexal surgeries or non-tubal reproductive surgeries without mentioning tubal reanastomosis and the above-mentioned outcomes were also excluded from our research.

### 2.4. Data Extraction

Full texts were independently reviewed by three reviewers (E.Z., N.P. and I.K.P.). For each study included, data were extracted where available on study design, patient characteristics, technical details, operative outcomes, reproductive outcomes, and economic outcomes. When outcome definitions differed across studies, the original study definitions were retained, and no cross-study recalculation was attempted. Data were synthesized descriptively without quantitative pooling.

### 2.5. Data Synthesis and Integration

Data were synthesized narratively and organized into the following four domains:

1. Characteristics of the evidence base.
2. Operative outcomes.
3. Reproductive outcomes and patient-selection factors.
4. Economic and counseling considerations.

Primary robotic clinical studies were summarized separately from secondary review-level or contextual publications to avoid conflation of direct and indirect evidence.

### 2.6. Methodological Appraisal and Evidence Level

Methodological appraisal was performed using design-appropriate instruments. Primary non-randomized clinical studies were assessed with the Methodological Index for Non-Randomized Studies (MINORS) [17]. Included systematic reviews and meta-analyses were appraised with AMSTAR 2 [18]. Narrative reviews and technical notes were retained for contextual interpretation but were not treated as primary effectiveness evidence and therefore were not formally risk-of-bias scored. Two reviewers performed the appraisal independently, and disagreements were resolved by consensus with a third reviewer. In addition, levels of evidence were assigned according to the Oxford Centre for Evidence-Based Medicine (OCEBM) framework [19]. Detailed appraisal results are presented in Supplementary File S1.

### 2.7. Ethical Considerations

Our review used existing data from previously published research, which did not require any institutional ethics board approval.

## 3. Results

### 3.1. Studies Included

Our research included sixteen peer-reviewed studies [7,9,10,11,12,13,14,15,16,20,21,22,23,24,25,26] which evaluated robotic-assisted tubal reanastomosis surgeries (Table 1). These contained:

1. Two early feasibility studies [9,10].
2. Three comparative cohort studies [7,20,25].
3. Six institutional cohort studies/case series [12,15,21,22,23,24].
4. Two systematic reviews and/or meta-analysis [14,26].
5. Two narrative reviews [13,16].
6. One technical-standardization report [11].

The primary evidence base consisted predominantly of retrospective single-center cohorts and case series, with limited comparative observational data, while secondary sources included systematic reviews/meta-analyses, narrative reviews, and technical standardization reports. These categories were interpreted separately throughout the manuscript to distinguish direct robotic clinical evidence from broader contextual or interpretive literature. No randomized controlled trials were identified.

Some publications may represent overlapping patient populations from the same centers and periods. Therefore, cumulative interpretation should be made cautiously, and the included studies should not be assumed to reflect entirely independent cohorts. In particular, the robotic patients reported by Goldberg and Falcone [20] correspond to the previously published pilot robotic cohort of Falcone et al. [10].

### 3.2. Operative Outcomes

Across the published robotic series, operative time appeared to decrease in later and higher-volume reports, although comparisons across studies should be interpreted cautiously because of differences in robotic platform generation, surgeon experience, case selection, and reporting methods [7,9,10,12,15,20,21,22,23,24,25]. Early experience with the ZEUS platform based on data from 10 cases reported a mean operative time of 284 ± 49.5 min [10]. In the corresponding comparative study by Goldberg and Falcone, the robotic arm retained the same operative-time profile because it represented the same previously published robotic cohort [20]. Degueldre et al. reported technical feasibility in eight patients using the da Vinci system, with all procedures completed robotically and all 16 tubes successfully reanastomosed [9]. Vlahos et al. later reported a mean operative time of 172 ± 53 min in a small teaching-institution series [21].

In comparative observational data, Rodgers et al. reported a median robotic surgical time of 229 min, which was longer than minilaparotomy, although blood loss, hospitalization, and pregnancy outcomes were favorable [7]. Patel et al. reported a mean robotic operative time of 201 min, again longer than open microsurgery, but with shorter hospitalization and comparable cost per delivery in their cohort [15]. Caillet et al. described a mean operative time of 110 ± 22.9 min in a larger retrospective series [22], whereas Göçmen et al. and Kavoussi et al. reported mean or median times of approximately 130–146 min in smaller later cohorts [23,24]. Ghomi et al. demonstrated a marked reduction in operative time within a single experienced program over time, from 140.7 ± 27.0 min in the first year to 60.0 ± 9.1 min in the final year of observation [12]. In the larger comparative study by Elci et al., mean robotic operative time was 214.72 ± 20.45 min [25].

All studies report minimal estimated blood loss when reported [7,10,21,24] and an absence of major intraoperative complications. No conversions to open surgery were documented. Minor complications were also rare and included a case of port-site infection [15], a case of a trocar injury of the inferior epigastric artery that was managed perioperatively with bipolar cautery [12], and one readmission due to tachycardia [7]. No transfusions, re-operations or RATR-related mortalities were reported. Length of hospital stay was typically less than 24 h. However, a few studies reported slightly longer hospitalization [23,25].

Overall, the operative literature suggests that RATR is technically feasible and associated with a favorable minimally invasive recovery profile in experienced hands. However, cross-study comparison remains limited by heterogeneous reporting and by differences in platform generation, center experience, and case selection. Table 2 summarizes operative characteristics of all published RATR cases after sterilization.

### 3.3. Reproductive Outcomes

Patency endpoints were generally encouraging when reported, but denominators differed substantially. Falcone et al. reported postoperative hysterosalpingography patency in 17 of 19 tubes (89.5%) [10]. In the later comparative report by Goldberg and Falcone, the same robotic cohort was reported with a similar patency figure [20]. Vlahos et al. documented patency in 7 of 8 tested tubes (87.5%) [21], while Kavoussi et al. reported at least unilateral patency in 16 of 17 evaluable patients (94.1%) [24]. Ghomi et al. reported tubal patency in 42 of 52 patients with available follow-up data (81%), again illustrating the denominator limitations imposed by incomplete follow-up [12]. In Degueldre et al., postoperative tubal patency was confirmed, but the cohort was small and the assessment strategy was not directly comparable with later patient-level reports [9].

Pregnancy outcomes ranged widely across studies. In the earliest feasibility series by Degueldre et al., two pregnancies occurred among eight women within four months of surgery (25%) [9]. Falcone et al. reported five pregnancies in 10 patients in their pilot robotic cohort [10], and Goldberg and Falcone later reported a 50% clinical pregnancy rate in the same robotic arm [20]. Vlahos et al. reported conception in four of five women (80%) within 12 months, although the cohort was very small [21]. Rodgers et al. documented 14 conceptions among 23 robotic patients with available follow-up, corresponding to 19 pregnancies in total [7]. Dharia Patel et al. reported a 62.5% pregnancy rate in the robotic group [15].

Larger cohorts provided more stable but still non-comparable estimates. Caillet et al. reported that 66 of 93 women achieved at least one pregnancy within two years (71%), with a median time to conception of 8.0 months; notably, 56% of pregnancies occurred within three months and 97% within 24 months [22]. Göçmen et al. reported pregnancy in 7 of 10 women (70%) [23]. Kavoussi et al. reported conception in 10 of 17 evaluable patients (58.8%), with a mean time to conception of 6 months [24]. Ghomi et al. reported pregnancy in 35 of 59 patients with available follow-up (59%) [12]. In the comparative multicenter study by Elci et al., 63 pregnancies were reported in the robotic group (61.2%), with a mean time to conception of 7.94 ± 3.79 months and no pregnancies after 15 months of follow-up [25].

Delivery/live-birth reporting was also heterogeneous. Vlahos et al. reported two live births in five women (40%) [21]. Göçmen et al. reported five live births in 10 women (50%) [22]. Caillet et al. reported that 58 of 93 women had at least one delivery within two years (62%) [22]. Elci et al. reported a live-birth rate of 46.6% in the robotic group [25]. By contrast, some studies reported viable intrauterine pregnancies or cost-per-delivery outcomes rather than directly comparable woman-level live-birth rates [7,15,24].

Ectopic pregnancy was an important reproductive safety endpoint. No ectopic pregnancy was reported in the earliest Falcone/Goldberg robotic cohort [10,20]. Vlahos et al. reported one ectopic pregnancy [21]. Rodgers et al. reported two ectopic pregnancies among 19 total pregnancies (11%) in the robotic group [7]. Patel et al. reported a higher abnormal pregnancy burden in the robotic arm, including four ectopic pregnancies and two spontaneous pregnancy losses [15]. Göçmen et al. reported one ectopic pregnancy (10%) [23], Kavoussi et al. one ectopic pregnancy (5.9%) [24], and Elci et al. three ectopic pregnancies (2.9%) [25].

Taken together, the reproductive literature suggests that pregnancy after RATR is feasible and may be clinically meaningful in selected women. However, the wide variation in reported rates reflects substantial heterogeneity in patient selection, follow-up, tube-level versus patient-level denominators, and outcome definitions. Table 3 summarizes reproductive outcomes of all published RATR cases after sterilization.

### 3.4. Patient Selection and Counseling Considerations

Age is consistently one of the strongest predictors of fertility after tubal reversal; however, counseling should not be based on age alone [2,26,27]. Post-reversal success is also influenced by residual tubal length, site of anastomosis, type of prior sterilization, interval since sterilization, ovarian reserve, prior reproductive history, male-factor infertility, and the presence of additional pelvic or uterine pathology [26,27].

These variables are incompletely and inconsistently reported in the robotic literature, which limits direct adjustment across studies. Nevertheless, the broader sterilization-reversal literature suggests that adequate remaining tubal length and favorable tubal anatomy are important predictors of pregnancy, whereas severe tubal damage, extensive excision, fimbrial loss, or multiple competing infertility factors may reduce the probability of success [14,26,27]. Previous reproductive performance and the presence of male-factor infertility should also be considered before recommending reversal over IVF [26,27].

For this reason, the choice between RATR and IVF should be framed as an individualized counseling decision rather than a binary or universally age-driven algorithm. In women with favorable anatomy, adequate tubal remnants, and no major competing infertility factors, surgical reversal may offer the possibility of repeated natural conception. Conversely, IVF may be preferable when tubal anatomy is unfavorable, ovarian reserve is reduced, male-factor infertility is present, or rapid conception is prioritized [26,27].

### 3.5. Economic Considerations

The available economic literature on robotic-assisted tubal reanastomosis is limited and should be interpreted cautiously. Published cost analyses are retrospective, institution-specific, and based on heterogeneous accounting methods, time periods, and comparators [7,15,26]. Available studies suggest that robotic procedures may involve higher upfront procedural costs than open or minilaparotomy reversal in some settings [7,15]. At the same time, cost-per-delivery estimates have been reported as similar to open microsurgery in selected cohorts, illustrating that immediate procedural cost and downstream reproductive cost are not necessarily equivalent [15].

Rodgers et al. found that robotic tubal anastomosis was more expensive than minilaparotomy in their institution, despite a favorable minimally invasive recovery profile [7]. Patel et al. reported higher direct procedural costs for robotics than for open microsurgery, but similar cost per delivery in their cohort [15]. Ghomi et al. later provided contemporary single-institution cost data in a mature robotic program, but these findings remain difficult to generalize beyond their local setting [12].

The current literature does not provide robust age-stratified cost-per-live-birth analyses for robotic reversal, nor does it allow contemporary head-to-head economic comparison with IVF across healthcare systems [26]. For these reasons, economic counseling should be framed conceptually rather than as a definitive comparative model. Recovery profile, institutional expertise, local cost structures, the possibility of multiple future natural conceptions after successful reversal, and the expected need for repeated IVF cycles may all influence real-world decision-making [26,27].

### 3.6. RATR vs. IVF

Available evidence suggests that both strategies are strongly age-dependent and that patient selection remains central [26,27]. RATR may be a reasonable fertility-restoration option in carefully selected women with favorable tubal anatomy and no major competing infertility factors, whereas IVF may be more appropriate when tubal anatomy is unfavorable, ovarian reserve is diminished, male-factor infertility is present, or rapid conception is clinically prioritized [26,27]. The potential advantage of successful reversal is that it may permit more than one spontaneous conception over time, whereas IVF is cycle-dependent [4,26,27]. However, this theoretical advantage must be weighed against age-related fertility decline, comorbid infertility factors, and the uncertainty introduced by non-standardized observational data.

Accordingly, the current literature supports individualized counseling rather than claims of equivalence or superiority between RATR and IVF.

### 3.7. Descriptive Historical Evolution

From the first feasibility reports in 2000 to more recent standardization-oriented publications, the RATR literature suggests progressive technical refinement and broader procedural maturity [9,10,11]. Early work focused on proof of concept and microsurgical feasibility with the ZEUS and early da Vinci systems [9,10,20]. Subsequent series from specialized centers provided more detailed reproductive outcomes and comparative observational data [7,15,21,22,23,24,25]. More recently, technical standardization reports, larger retrospective cohorts, and comparative multicenter data have strengthened the descriptive literature [11,12,25].

However, these observations should be interpreted descriptively, not as formal longitudinal trend analyses, because the available studies differ markedly in design, case selection, platform generation, outcome definitions, and follow-up. The chronological evolution of operative performance, reproductive outcomes and procedural refinement in RATR is summarized in Table 4.

## 4. Discussion

### 4.1. Interpretation of the Current Evidence Base

This review suggests that RATR is technically feasible and may provide clinically meaningful reproductive outcomes in selected women after sterilization. Across the available series, perioperative morbidity was low, recovery was generally consistent with minimally invasive surgery, and pregnancy after robotic reversal was repeatedly documented [7,9,10,12,15,20,21,22,23,24,25]. At the same time, the quality and structure of the evidence remain important limiting factors.

The direct robotic literature is composed predominantly of retrospective single-center cohorts and small series, with only limited prospective data and no randomized trials [12,14,26]. Even among the larger cohorts, outcome ascertainment is often incomplete, denominators differ across endpoints, and patient-selection factors are incompletely reported [12,22,25]. These issues make quantitative comparison across studies inherently uncertain.

The reproductive results reported in the robotic literature are encouraging, but they should not be interpreted as definitive proof of equivalence with other reversal approaches or with IVF. Broader sterilization-reversal reviews suggest that surgery can offer good results in selected women, especially when favorable anatomy is present [14,26]. However, those broader reviews often include mixed approaches and cannot be treated as direct robotic-only evidence. The same caution applies when interpreting the relative place of robotic, laparoscopic, and open reversal in current practice [3,13,14,26].

### 4.2. Counseling in the IVF Era

The current IVF era has changed the way sterilization reversal is discussed, but it has not rendered tubal surgery obsolete [4,14,26,27]. IVF provides a highly standardized treatment pathway and may be particularly attractive when ovarian reserve is declining, tubal anatomy is poor, male-factor infertility is present, or rapid conception is a priority [5,6,17]. Conversely, successful reversal may offer the possibility of repeated natural conception without repeated treatment cycles [26,27].

This distinction is clinically important, but it should not be simplified into a binary choice between a “surgical” and an “ART” pathway. The appropriate counseling framework is individualized. Tubal length, sterilization method, anastomosis site, age, ovarian reserve, prior fertility, and additional infertility factors all influence prognosis [26,27]. In practice, the decision depends on the interaction of these variables rather than on age alone.

### 4.3. Indications for RATR Versus IVF After Sterilization

Counseling after sterilization should begin with a structured assessment of infertility factors, anatomic feasibility, patient age and ovarian reserve, and patient preferences (e.g., desire for natural conception, acceptable time to pregnancy, and family-building goals). Based on available guidance and synthesis in the reversal literature, RATR is most plausibly considered in women with favorable anatomic prerequisites (including adequate expected residual tubal length, commonly >4 cm), absence of major pelvic adhesions or advanced endometriosis, and no significant additional infertility factors, particularly when the patient prioritizes natural conception and potentially multiple future pregnancies and when experienced robotic reproductive surgeons and appropriate infrastructure are available [13,26].

IVF is generally preferred when anatomic reconstruction is unlikely to yield timely success or is not feasible. This includes advanced maternal age (commonly >40 years), diminished ovarian reserve, insufficient residual tubal length or severely compromised tubal anatomy precluding reconstruction, male-factor infertility or other additional infertility factors, and situations in which rapid time-to-pregnancy is a priority because IVF bypasses the need for natural-cycle conception [13,26].

These strategies should not be framed as universally competing alternatives. In appropriate candidates, RATR can restore the possibility of natural conception following a single surgical intervention, whereas IVF provides a controlled and time-sensitive pathway that can bypass unfavorable anatomy and address coexisting infertility factors [13,26]. When both options are available, individualized selection and transparent counseling are required to optimize clinically meaningful outcomes.

### 4.4. Operative and Technical Perspective

RATR appears to be a technically suitable minimally invasive adaptation of microsurgical tubal reconstruction. The robotic platform may be particularly attractive for reconstructive procedures that require precise dissection and suturing in confined spaces [8,9,10,11,12]. However, the present review does not justify broad claims that robotics is universally superior to conventional laparoscopy or open microsurgery for all candidates. The available evidence is too limited and too heterogeneous to support such conclusions [7,15,20,25].

Similarly, although later and higher-volume reports suggest improved operative efficiency over time, this should be interpreted as a descriptive observation rather than proof of a universally shorter learning curve. Operative time is influenced by multiple factors, including robotic generation, prior surgical experience, center volume, complexity of tubal damage, and reporting practices [7,12,22,25].

Broader gynecologic robotics literature confirms that robotic surgery has become an established part of gynecologic practice and may offer procedural advantages in selected settings [28,29]. However, evidence from broader gynecologic robotics should be interpreted as contextual rather than procedure-specific evidence for tubal reanastomosis.

### 4.5. Economic and Health-System Considerations

The economic question remains unresolved. The currently available robotic cost studies are limited by retrospective design, institutional accounting methods, and small cohorts [7,15]. Although robotics may increase direct procedural cost in some settings, this does not automatically determine the cost-effectiveness of reversal as a fertility strategy, because real-world counseling also depends on the probability of repeated natural conception, the expected need for multiple IVF cycles, and the local structure of healthcare reimbursement [26,27].

The economic literature is suggestive but not definitive. The present evidence does not justify universal claims that RATR is broadly cost-effective or broadly cost-ineffective compared with IVF across health systems.

### 4.6. Future Directions

Future research should focus on prospective multicenter studies with standardized reporting of operative outcomes, tubal patency, pregnancy definitions, live birth, ectopic pregnancy, and follow-up duration. Comparative evaluation against other reversal approaches and against IVF will require better control of baseline prognostic factors, including age, tubal anatomy, ovarian reserve, and male-factor infertility [27]. More robust and contemporary economic analyses are also needed across different healthcare settings.

Emerging technological developments such as improved haptic feedback and artificial-intelligence-assisted robotic systems may influence future microsurgical platforms, although their specific relevance to tubal reanastomosis remains speculative at present [30,31]. Although published literature on tubal reanastomosis suggests that experimental adjunctive concepts such as fibrin sealants achieve better results when used as adjuncts to microsuturing [32,33], they remain investigational and were not part of the structured clinical evidence base of the present review.

### 4.7. Limitations of the Current Evidence Base

The majority of RATR publications show strong cumulative evidence yet they use retrospective studies and single-institution case series as their primary research approach [12,22,23,24,25]. The current research lacks direct randomized controlled trials that compare RATR to IVF. Sample heterogeneity together with inconsistent reporting of essential metrics such as tubal length, male-factor infertility and previous sterilization techniques limit meta-analytic strength. Follow-up duration is also heterogeneous, and reproductive outcome definitions are not uniform, which limits direct comparison across reports.

In addition, some publications may reflect overlapping patient populations from specialized centers, and publication bias toward favorable surgical outcomes cannot be excluded [10,20]. Comparative assessment against IVF is indirect, because IVF data are generally derived from registry-based datasets rather than contemporary matched cohorts [5,6,26,27,34]. Economic evaluations are similarly limited by retrospective design, institution-specific costing, and the absence of standardized age-stratified cost-per-live-birth models [7,15,26].

## 5. Conclusions

Available evidence suggests that robotic-assisted tubal reanastomosis is a feasible minimally invasive option for fertility restoration in carefully selected women after sterilization. Reported reproductive outcomes appear encouraging and, in selected series, broadly comparable to those reported for other surgical reversal approaches. However, the evidence base remains limited by retrospective design, small sample size, heterogeneous outcome reporting, variable follow-up, and the absence of direct contemporary comparisons with IVF.

Accordingly, RATR should be considered as one component of individualized fertility counseling rather than as a universal substitute for IVF. Future prospective comparative studies with standardized outcome definitions, transparent reporting of prognostic factors, and robust economic evaluation are needed to better define its role in modern reproductive practice.

## Figures and Tables

**Table 1:** (**A**) Primary clinical studies of robotic-assisted tubal reanastomosis included in the review. (**B**) Secondary and contextual publications retained for narrative synthesis.

**(A)**				
**Authors (Year)**	**Study Design**	**Platform**	**N (Patients)**	**Key Findings—Notes**
Degueldre et al. (2000) [9]	Descriptive feasibility case series	da Vinci	8	First RATR using da Vinci surgical system; technical feasibility; 90% tubal patency
Falcone et al. (2000) [10]	Feasibility pilot study	ZEUS	10	First human robotic-assisted cohort; 89.5% patency; 5 pregnancies within follow-up time (12 months); no conversions
Goldberg & Falcone (2003) [20]	Retrospective comparative case study (robotic vs. conventional laparoscopy)	ZEUS	Robotic arm = 10 (robotic group same as Falcone et al. (2000) [10]) Laparoscopic arm = 15	Robotic: longer operative time; Robotic group: patency rate 89.5%, pregnancy rate within first year 50%. All pregnancies delivered. No complications reported
Vlahos et al. (2007) [21]	Case series	da Vinci	5	1 patient conceived after second menstrual cycle post-op. The remaining 4 underwent HSG and 7 out of 8 tubes were patent (87.5% patency rate); 80% pregnancy rate; 40% live-birth rate; 1 ectopic pregnancy; 1 biochemical pregnancy; mean time to conception 5.5 ± 2 months
Rodgers et al. (2007) [7]	Retrospective case–control study (robotic vs. minilaparotomy)	da Vinci	Robotic arm = 26 Minilaparotomy arm = 41	Comparable fertility; higher robotic cost; faster recovery; robotic group: 19 pregnancies achieved in 14 patients; 11% ectopic pregnancy rate; 26% spontaneous abortion rate (lower than laparotomy group)
Patel et al. (2008) [15]	Prospective cohort study (robotic vs. open laparotomy)	da Vinci	Robotic arm = 18 Open laparotomy arm = 10	Cost-per-live-birth analysis; Comparable pregnancy rates; similar cost per live birth; robotic group: pregnancy rate 62.5%; 4 ectopic pregnancies; 1 spontaneous pregnancy loss
Caillet et al. (2010) [22]	Retrospective cohort study	da Vinci	97 with available follow-up	Largest early single-center robotic cohort; age-stratified fertility; 71% pregnancy; 62% live birth; for each increase in age of 5 years, the probability of becoming pregnant decreases by 38%
Göçmen et al. (2013) [23]	Retrospective case series	da Vinci	10	70% pregnancy rate; no conversions
Kavoussi et al. (2014) [24]	Retrospective case series	da Vinci	18	One-stitch technique; 94.1% tubal patency
Ghomi et al. (2020) [12]	Retrospective cohort study	da Vinci	106	Learning curve reduced operative time; 59% pregnancy; 81% patency
Elci et al. (2022) [25]	Retrospective comparative multicenter study (laparotomy vs. laparoscopy vs. robotic)	da Vinci	Robotic arm = 103 Laparoscopic arm = 55 Laparotomy arm = 78	No statistical difference in pregnancy rates among approaches; pregnancy rates were very high in all groups
(**B**)				
**Authors (Year)**	**Study Design**	**Platform**	**N (Patients)**	**Notes—Key Findings**
Netter et al. (2023) [11]	Technical note (step-by-step surgical standardization)	da Vinci	1	10-step standardized robotic protocol
Salehjawich et al. (2022) [16]	Narrative review (technique and family planning focus)	da Vinci	Not applicable	Technique-focused review; Supports robotic approach; discusses technical and counseling aspects
Madison et al. (2021) [13]	Narrative review	Mixed (microsurgery vs. laparoscopic vs. robotic discussed)	Not applicable	Secondary opinion-based synthesis. Laparoscopy favored <40 yrs
van Seeters et al. (2017) [26]	Systematic review	Mixed (including robotic)	10,689 (37 studies)	Contextualizes broader sterilization-reversal evidence, prognostic factors, and indirect comparison with IVF. Pregnancy 42–69%; age strongest predictor; no superiority of robotic approach
Sastre et al. (2023) [14]	Systematic review & meta-analysis	Mixed (including robotic)	14,113 (22 studies)	Includes mixed surgical approaches and broad study designs. Overall pregnancy 65%; no difference among surgical approaches

Abbreviations: RATR: robotic-assisted tubal reanastomosis, HSG: hysterosalpingography. IVF: in vitro fertilization.

**Table 2:** Operative characteristics of published primary robotic clinical studies ^1^.

Authors (Year)	Robotic System	N (Patients)	Mean Total Operative Time (min)	Blood Loss (mL)	Mean Hospital Stay	Conversion to Laparotomy	Complications
Falcone et al. (2000) [10]	ZEUS	10	284 ± 49.5	70 ± 68 mL	<24 h	None	None
Degueldre et al. (2000) [9]	da Vinci	8	181.5 ± 42.6	NR	1.5 days	None	None
Goldberg & Falcone (2003) [20] (robotic arm)	ZEUS	10 (same patients as Falcone et al. [10])	284 ± 49.5	70 ± 68 mL	<24 h	None	None
Vlahos et al. (2007) [21]	da Vinci	5	172 ± 53	minimal	<24 h	None	None
Rodgers et al. (2007) [7] (robotic arm)	da Vinci	26	229 median total time (range 205–252)	73% less than 100 mL	<3 h	None	1 readmission due to tachycardia
Patel et al. (2008) [15] (robotic arm)	da Vinci	18	201 (range 140–263)	NR	4 h	None	1 trocar injury to the inferior epigastric artery (controlled perioperatively)
Caillet et al. (2010) [22]	da Vinci	97	110 ± 22.9	NR	NR	None	NR
Göçmen et al. (2013) [23]	da Vinci	10	130.6 (range 102–164)	NR	1.2 days	None	None
Kavoussi et al. (2014) [24]	da Vinci	18	146 (range 89–203)	<25 mL	Same-day discharge	None	None
Ghomi et al. (2020) [12]	da Vinci	106	140 ± 27 (first year of the study); 60 ± 9.1 (last year of the study)	NR	Outpatient	None	1 port-site infection
Elci et al. (2022) [25] (robotic arm)	da Vinci	103	214.72 ± 20.45	NR	1.74 ± 0.47 days	None	None

Abbreviations: NR: not reported. ^1^ Only primary clinical studies reporting robotic-specific operative data were included. In comparative studies, only robotic arm data are presented. Complications include intraoperative and early postoperative events explicitly reported.

**Table 3:** Reproductive outcomes following robotic-assisted tubal reanastomosis ^1^.

Authors (Year)	N (Patients)	Follow-Up (Months)	Tubal Patency	Pregnancy Outcome	Live-Birth/Delivery Outcome	Ectopic Pregnancy Outcome	Notes
Falcone et al. (2000) [10]	10	12	89.5%	5 pregnancies	50%	0%	Patency diagnosed with hysterosalpingograms postoperatively
Degueldre et al. (2000) [9]	8	4	90%	25%	NR	None reported within 4 months	2 pregnancies occurred within 4 months. 5 out of 8 patients underwent hysterosalpingography 3 months post-op and tubal patency was calculated.
Goldberg & Falcone (2003) [20] (robotic arm)	10	>12	89.5%	50%	50%	0%	All pregnancies occurred within 12 months
Vlahos et al. [21] (2007)	5	12	87.5% (7/8 tubes tested patent)	80% (4/5 women conceived)	40% (2/5 women had live births)	1 ectopic pregnancy (20% of women; 25% of pregnancies)	Mean time to conception: 5.5 months; also reported one biochemical pregnancy; denominators are mixed
Rodgers et al. [7] (2007) (robotic arm)	26 total; 23 with pregnancy follow-up	>10	NR	14/23 women conceived (61%); 19 pregnancies total	14/19 pregnancies (74%) were viable intrauterine pregnancies (ongoing or delivered)	2/19 ectopic pregnancies (11%)	Median time to conception: 2 months (range 0–9 months); 14 patients conceived 19 times.
Patel et al. [15] (2008) (robotic arm)	18	8.9	NR	62.5%	28%	22% (4 ectopic pregnancies)	Two spontaneous pregnancy losses also reported
Caillet et al. [22] (2010)	97	24	NR	71% (66/93 women had at least one pregnancy within 2 years)	62% (58/93 women had at least one delivery within 2 years)	NR	56% of the pregnancies occurred within 3 months and 97% within 24 months after surgery; 8 patients gave birth twice
Göçmen et al. [23] (2013)	10	18	NR	70%	50%	10%	All pregnancies occurred within a year; 1 spontaneous pregnancy losses
Kavoussi et al. [24] (2014)	18 total; 17 evaluable	NR	94.1% (16/17 patients had at least one tube patent)	58.8%	41.2% (7/17 intrauterine pregnancies with fetal cardiac motion)	5.9% (1/17 ectopic pregnancy)	Mean time to conception: 6 months (range 2–22); 2 spontaneous pregnancy losses
Ghomi et al. [12] (2020)	106	37.5	81% (42/52 patients with available data)	59% (35/59 patients with available data conceived)	NR	NR	Strong denominator heterogeneity because of dropout; age-stratified pregnancy also reported
Elci et al. [25] (2022) (robotic arm)	103	24	HSG performed in 14% of robotic non-pregnant subgroup after 1 year; no global patency rate reported	61.2% (63/103 pregnancies)	46.6% (48/103 live births)	2.9% (3/103 ectopic pregnancies)	Mean time to conception: 7.94 ± 3.79 months; all pregnancies occurred within 15 months after surgery; 11.7% postoperative abortion rate

Abbreviations: NR: not reported, HSG: hysterosalpingography. ^1^ Only primary clinical studies reporting robotic-arm-specific reproductive outcomes were included. In comparative studies, only robotic arm data are presented. Follow-up duration is listed only when explicitly stated in the manuscript. Ectopic rate reflects reported ectopic pregnancies within the robotic cohort. Outcome definitions and denominators differ across studies.

**Table 4:** Historical observations in robotic-assisted tubal reanastomosis ^1^.

	Early Era	Intermediate Era	More Recent Era	Interpretation
**Robotic Platform**	ZEUS/early da Vinci	da Vinci Systems	da Vinci Xi	Platform maturation likely contributed to procedural refinement
**Operative Time**	Falcone/Goldberg-era robotic times around 284 min	Göçmen and Kavoussi reported 130–146 min; Caillet 110 ± 22.9 min	Ghomi reported reduction from 140.7 ± 27.0 min to 60.0 ± 9.1 min within one program	Efficiency appears to improve over time, but comparisons remain non-standardized
**Hospital Stay**	Mostly <24 h in early series	1–2 days in some centers	Outpatient/same-day discharge in some later reports	Minimally invasive recovery profile is consistent, but local practice varies
**Tubal Patency Reporting**	Early focus on technical feasibility and HSG patency	High patency reported in selected series	Not routinely reported	Patency data are encouraging but inconsistently measured
**Pregnancy Reporting**	Small early studies with short follow-up	Larger cohorts begin to provide age-stratified fertility outcomes	Small early studies with short follow-up	Pregnancy outcomes remain difficult to compare because of different denominators and follow-up
**Economic Reporting**	Sparse	Comparative cost studies appear	Contemporary single-center cost reporting re-emerges	Cost evidence remains institution-specific and not generalizable
**Technical Refinement**	Feasibility and basic robotic microsuturing	One-stitch adaptations and step standardization emerge	Mature center-specific optimization reported	Technical standardization increased over time

^1^ These are descriptive observations only, not formal longitudinal trend analyses. Study designs, case selection, follow-up, and outcome definitions differ substantially across eras.

## Data Availability

No new data were created or analyzed in this study.

## Supplementary Materials

The following supporting information can be downloaded at: https://www.mdpi.com/article/10.3390/medicina62061054/s1, File S1: Search Strategy, Methodological Quality and Evidence Level of Included Studies.

## Abbreviations

The following abbreviations are used in this manuscript:

ART	Assisted Reproductive Technologies
RATR	Robotic-assisted Tubal reanastomosis
IVF	In Vitro Fertilization
HSG	Hysterosalpingography
MINORS	Methodological Index for Non-Randomized Studies
OCEBM	Oxford Centre for Evidence-Based Medicine
