# Supplementary material for: Robotic-Assisted Tubal Reanastomosis After Sterilization in the IVF Era: A Narrative Review
Source: Medicina (Kaunas). 2026 May 29;62(6):1054. doi: 10.3390/medicina62061054 (PMC13304470; doi:10.3390/medicina62061054)
Supplement: Supplementary file 1 [file medicina-62-01054-s001.zip › medicina-4328359-supplementary.pdf]

Search Strategy

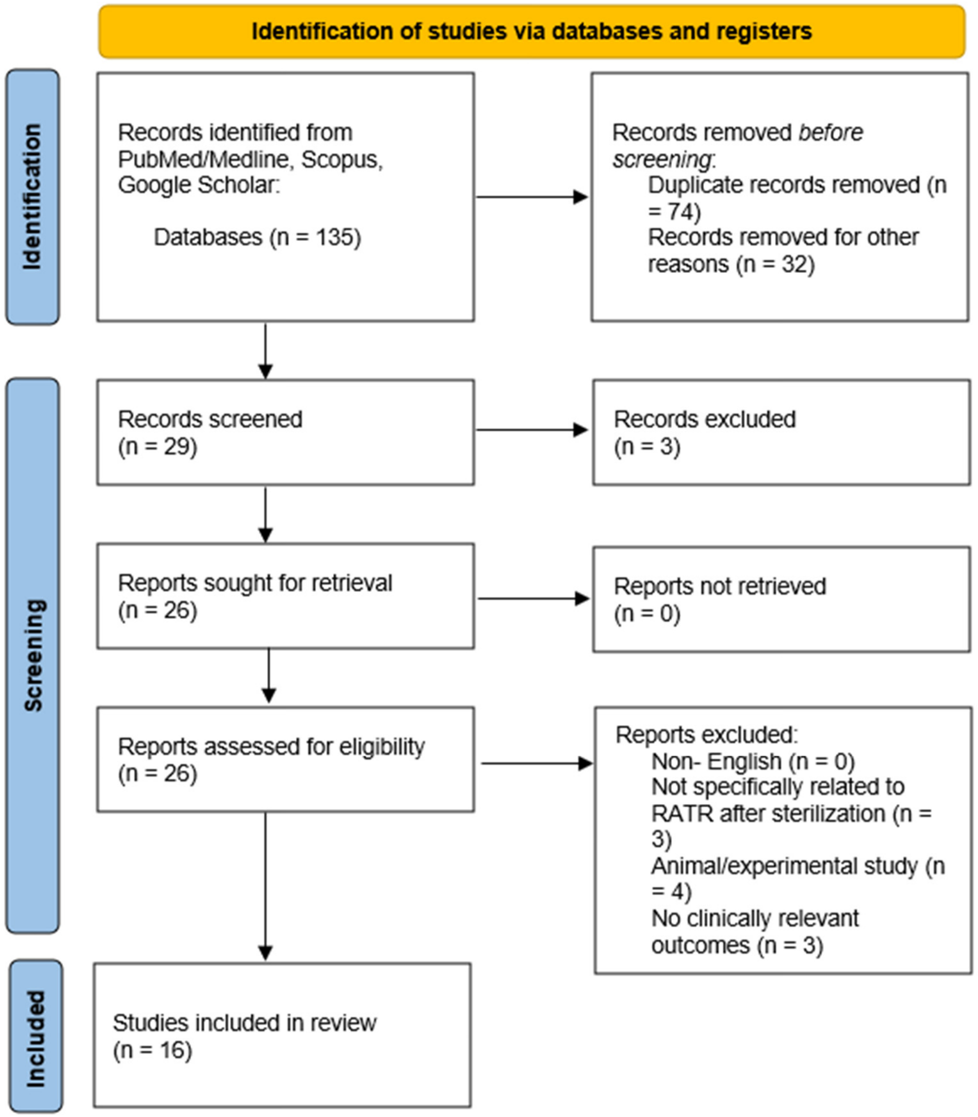

Supplementary Figure S1. PRISMA flowchart of the included studies.

Methodological Quality and Evidence Level of Included Studies

Supplementary Table S1. MINORS item legend.

MINORS item	Description
**1**	Clearly stated aim
**2**	Inclusion of consecutive patients
**3**	Prospective collection of data
**4**	Endpoints appropriate to the aim of the study
**5**	Unbiased assessment of the study endpoint
**6**	Follow-up period appropriate to the aim of the study
**7**	Loss to follow-up less than 5%
**8**	Prospective calculation of the study size
**9**	Adequate control group*
**10**	Contemporary groups*
**11**	Baseline equivalence of groups*
**12**	Adequate statistical analyses*

Scoring: 0 = not reported; 1 = reported but inadequate; 2 = reported and adequate. Maximum score: 16 for non-comparative studies; 24 for comparative studies. *Items 9–12 apply only to comparative studies.

Supplementary Table S2. MINORS appraisal of non-comparative primary clinical studies.

Study	1	2	3	4	5	6	7	8	Total /16	Brief interpretation
**Degueldre et al. (2000) [9]**	2	0	2	2	1	1	1	0	9	Early feasibility study with prospective conduct and objective patency assessment, but short fertility follow-up and unclear consecutive inclusion/attrition reporting
**Falcone et al. (2000) [10]**	2	0	2	2	1	1	2	0	10	Prospective pilot study with clear operative and patency endpoints; follow-up was still early and no sample-size justification was provided
**Vlahos et al. (2007) [21]**	2	0	1	2	1	2	2	0	10	Small case series with complete short-term follow-up and objective postoperative assessment, but limited methodological reporting and no explicit prospective protocol statement
**Caillet et al. (2010) [22]**	2	1	0	2	1	2	0	0	8	Large retrospective reproductive cohort with meaningful time-to-pregnancy analysis, but substantial loss to follow-up and retrospective design limit internal validity
**Göçmen et al. (2013) [23]**	2	2	1	2	1	1	0	0	9	First 10 consecutive patients were reported, but follow-up was short and incomplete, with acknowledged difficulties in long-term outcome capture

Study	1	2	3	4	5	6	7	8	Total /16	Brief interpretation
**Kavoussi et al. (2014) [24]**	2	0	0	2	2	1	0	0	7	Retrospective patency-focused series with objective endpoint definition, but one patient was lost to follow-up and fertility follow-up was not uniformly structured
**Ghomi et al. (2020) [12]**	2	2	0	2	1	2	0	0	9	Contemporary consecutive cohort with clear aims and outcomes, but retrospective design and substantial follow-up dropout affect pregnancy and patency estimates

Supplementary Table S3. MINORS appraisal of comparative primary clinical studies.

Study	1	2	3	4	5	6	7	8	9	10	11	12	Total /24	Brief interpretation
**Goldberg and Falcone (2003) [20]**	2	1	0	2	1	2	0	0	2	1	1	2	14	Sequential robotic-versus-conventional laparoscopic comparison with appropriate statistics, but groups were not fully balanced and the robotic arm was the previously published pilot cohort
**Rodgers et al. (2007) [7]**	2	2	0	2	1	2	0	0	2	2	2	2	17	Stronger retrospective comparative design using all cases from the same period with comparable baseline demographics, but outcome follow-up was incomplete

Study	1	2	3	4	5	6	7	8	9	10	11	12	Total /24	Brief interpretation
**Patel et al. (2008) [15]**	2	1	2	2	1	1	1	0	2	2	1	2	17	Prospective comparative cohort with clinical and economic outcomes, but small size, non-random allocation, and unequal follow-up reduce certainty
**Elci et al. (2022) [25]**	2	1	0	2	1	2	0	0	2	2	1	2	15	Large retrospective three-arm comparison with explicit exclusion criteria and adequate statistics, but retrospective selection and some baseline imbalance remain important limitations

Supplementary Table S4. AMSTAR 2 appraisal of included systematic reviews and meta-analyses.

Review	Protocol registered before conduct	Search strategy adequate	Study selection / review process in duplicate	List of excluded studies provided	Risk of bias assessment of included studies	Meta-analytic methods appropriate	Risk of bias considered in interpretation	Publication bias assessed	Conflict of interest reported	Overall AMSTAR 2 confidence	Brief interpretation
**van Seeters et al. (2017) [26]**	No clear prior protocol/registration reported	Yes	Not clearly reported	No full excluded-studies list	No formal validated risk-of-bias tool; STROBE-based quality approach used	Yes	Partially	Not clearly reported	Yes	Low	Methodologically transparent systematic review with clear searches and flow diagram, but critical domains are weakened by lack of protocol registration and absence of a formal validated risk-of-bias tool
**Sastre et al. (2023) [14]**	No clear prior protocol/registration reported	Partly adequate	Review process by two authors reported; duplicate extraction not fully detailed	No full excluded-studies list	No formal validated risk-of-bias tool; broad internal-validity assessment only	Yes	Partially	Not clearly reported	Yes	Critically low	Recent systematic review/meta-analysis with PRISMA framing and pooled estimates, but methodological confidence is reduced by limited search breadth, broad inclusion of heterogeneous designs, and absence of formal risk-of-bias assessment

Supplementary Table S5. Oxford Centre for Evidence-Based Medicine (OCEBM) level of evidence.

Study (Author, Year)	Study Design	Comparator	Sample Size	Follow-up Adequacy (≥6 months)	Outcome Reporting Completeness	OCEBM Level of Evidence
**Degueldre et al. 2000 [9]**	Prospective feasibility case series	None	8	Yes	Limited (operative only)	Level 4
**Falcone et al. 2000 [10]**	Prospective pilot cohort	None	10	No	Operative + reproductive	Level 4
**Goldberg et al. 2003 [20]**	Retrospective comparative cohort	Laparoscopy	25 total	Yes	Operative + partial reproductive	Level 3
**Vlahos et al. 2007 [21]**	Prospective case series	None	5	Yes	Operative + reproductive	Level 4
**Rodgers et al. 2007 [7]**	Retrospective case–control	Minilaparotomy	67 total	Yes	Operative + reproductive	Level 3
**Patel et al. 2008 [15]**	Prospective comparative cohort	Open microsurgery	28 total	Yes	Operative + reproductive + economic	Level 2b
**Caillet et al. 2010 [22]**	Retrospective cohort	None	97	Yes	Reproductive detailed	Level 4
**Göçmen et al. 2013 [23]**	Retrospective case series	None	10	Yes	Operative + reproductive	Level 4
**Kavoussi et al. 2013 [24]**	Retrospective case series	None	18	N/A	Patency-focused	Level 4

**Ghomi et al. 2020 [12]**	Retrospective cohort	None	109	Yes	Operative + reproductive	Level 4
**Elci et al. 2022 [25]**	Retrospective multicenter comparative cohort	Open + Laparoscopic	236 total	Yes	Operative + reproductive	Level 3
**Salehjawich et al. 2022 [16]**	Narrative review	N/A	N/A	N/A	Technique-focused	Level 5
**Madison et al. 2021 [13]**	Narrative review	N/A	N/A	N/A	Comparative synthesis	Level 5
**van Seeters et al. 2017 [26]**	Systematic review	Surgical approaches	10,689	N/A	Reproductive outcomes	Level 1a
**Sastre et al. 2023 [14]**	Systematic review & meta-analysis	Surgical approaches	14,113	N/A	Pooled outcomes	Level 1a
**Netter et al. 2023 [11]**	Technical standardization report	None	NR	No	Procedural description only	Level 5

According to the Oxford Centre for Evidence-Based Medicine criteria, the included literature comprises predominantly Level 3–4 observational cohort studies, complemented by Level 1a systematic reviews and meta-analyses, with isolated Level 2b prospective comparative data. No randomized controlled trials were identified.
